# Endoplasmic reticulum, Golgi, and lysosomes are disorganized in lung fibroblasts from chronic obstructive pulmonary disease patients

**DOI:** 10.14814/phy2.13584

**Published:** 2018-02-27

**Authors:** Julie Weidner, Linnea Jarenbäck, Ida Åberg, Gunilla Westergren‐Thorsson, Jaro Ankerst, Leif Bjermer, Ellen Tufvesson

**Affiliations:** ^1^ Department of Clinical Sciences Lund, Respiratory Medicine and Allergology Lund University Lund Sweden; ^2^ Department of Experimental Medical Science Lung Biology Lund University Lund Sweden

**Keywords:** Chronic obstructive pulmonary disease, endoplasmic reticulum, endoplasmic reticulum stress response, Golgi, lung fibroblast

## Abstract

Chronic Obstructive Pulmonary Disease (COPD) is often caused by smoking and other stressors. This causes oxidative stress, which induces numerous changes on both the transcriptome and proteome of the cell. We aimed to examine if the endomembrane pathway, including the endoplasmic reticulum (ER), Golgi, and lysosomes, was disrupted in fibroblasts from COPD patients as opposed to healthy ever‐smokers or never‐smokers, and if the response to stress differed. Different cellular compartments involved in the endomembrane pathway, as well as mRNA expression and apoptosis, were examined before and after the addition of stress in lung fibroblasts from never‐smokers, ever‐smokers, and patients with COPD. We found that the ER, Golgi, and lysosomes were disorganized in fibroblasts from COPD patients under baseline conditions. After a time course with ER stress inducing chemicals, changes to the phenotypes of cellular compartments in COPD patient fibroblasts were observed, and the expression of the ER stress‐induced gene *ERP72* was upregulated more in the COPD patient's cells compared to ever‐smokers or never‐smokers. Lastly, a tendency of increased active Caspase‐3 was observed in COPD fibroblasts. Our results show that COPD patients have phenotypic changes in the lung fibroblasts endomembrane pathway, and respond differently to stress. Furthermore, these fibroblasts were cultured for several weeks outside the body, but they were not able to regain proper ER structure, indicating that the internal changes to the endomembrane system are permanent in smokers. This vulnerability to cellular stress might be a cause as to why some smokers develop COPD.

## Introduction

Chronic obstructive pulmonary disease (COPD) is one of the leading causes of death world‐wide. Although the disease can be developed via a variety of different factors, such as occupational, environmental, and genetic alterations, the most prevalent cause of COPD is cigarette smoking (Tuder and Petrache 2012). It has been shown that cigarette smoking causes oxidative stress to the cells of the lungs (Tuder et al. 2003).

Stress is also a natural occurring incidence for all cells in the body. Whether this stress is due to a foreign chemical, heat stress, starvation, or oxidative stress, the cell must be able to quickly adapt to its current situation or else die. In order to adapt to stressors, signaling pathways in eukaryotic cells are initiated that allow for a cascade of genes and proteins to be activated, with the ultimate goal to be maintenance of homeostasis (Cao and Kaufman 2014; Schwarz and Blower 2016).

The endoplasmic reticulum (ER) is a central component in eukaryotic cells, responsible for protein translation, folding, and transport (Schwarz and Blower 2016). It is a continuous membrane system consisting of tubules and sheets that begin at the nuclear membrane and extend throughout the cell. Despite its structure, the ER is a dynamic organelle that continuously changes based on the state of the cell, and is at the beginning of the eukaryotic endomembrane system. Proteins are translated in the ER, where they can then be modified and packaged into vesicles bound for the Golgi complex. Through the Golgi, transported proteins can be further modified before sorting to their final localization at the lysosome or plasma membrane where the proteins function.

Due to its important and central role in the cell, the ER has several mechanisms to ensure that homeostasis is maintained. The ER stress response activates a cascade of signals that allows the cell to respond to unwanted stressors (Cao and Kaufman 2014). In eukaryotic cells, there are three major ER stress pathways that detect increased levels of unfolded proteins indicative of cellular stress. The three branches of the ER stress response pathway include via inositol requiring enzyme‐1 (IRE1), protein kinase‐like ER kinase (PERK), and activating transcription factor 6 (ATF6), which activate the upregulation of different genes involved in coping with the ER stress response and restoring homeostasis to the cell (Sano and Reed 2013).

Short‐term studies in mice on the oxidative stress present in the lungs have been examined (Geraghty et al. 2011; Kenche et al. 2013; Lee et al. 2016), but little is known about how long‐term smoking affects the cells of the lung, for example as in COPD, and if ER stress response is maintained when under continuous oxidative stress.

We aimed to examine if the endomembrane pathway was altered in patients with COPD as opposed to never‐smokers or healthy ever‐smokers. By using lung fibroblasts from these three subject groups, we observed the phenotypes of the endomembrane system under stressed and unstressed conditions. Furthermore, gene expression of four different genes previously stated to be induced under stress conditions (Oslowski and Urano 2011) was measured. Finally, we aimed to examine changes in active Caspase‐3 levels to determine if apoptosis was affected in these cells.

## Materials and Methods

### Subjects

Material from a total of six healthy never‐smokers, four healthy ever‐smokers, and fifteen COPD patients was used in the different experiments in this study (Table 1). COPD patients were defined according to the GOLD criteria (FEV_1_/FVC < 0.7). The subjects underwent bronchoscopy and central airway lung biopsies were obtained. Fibroblasts were grown out from biopsies in Dulbecco's modified eagle medium (DMEM) supplemented with 10% fetal calf serum as previously described (Tufvesson et al. 2011). Cells were harvested for analysis at passages 3–6. All subjects signed written informed consent and the study was approved by the Regional Ethics Review Board in Lund.

**Table 1:** Subject characteristics

	Never‐smokers (*n* = 6)	Ever‐smokers (*n* = 4)	COPD (*n* = 15)
Gender (Female/Male)	4/2	2/2	6/9
Age (years)	50 (42–57)	66 (65–67)	65 (60–70)
Smoking status (current/former)	0/0	0/4	8/7
Pack years	0	27 (23–33)	40 (27–45)
FEV_1_ (L)	3.44 (3.20–3.56)	2.97 (2.21–3.60)	1.63 (1.44–2.18)
FEV_1_ (%p)	114 (95–139)	91 (89–96)	55 (47–65)
FEV_1_/FVC	0.84 (0.81–0.88)	0.79 (0.75–0.82)	0.49 (0.44–0.55)

### Induction of ER stress

Time course experiments were performed over six hours. Fibroblasts were grown to confluency in six well plates for subsequent RNA extraction and qPCR analysis or in chambered slides for immunofluorescence staining. DMEM (supplemented with 10% fetal calf serum) containing: Tunicamycin (5 *μ*g/mL), Thapsagargin (1μM), Brefeldin A (50 *μ*mol/L), dithiothreitol (DTT, 1 mmol/L), or controls of EtOH (0.01%) or 1 × Phosphate Buffered Saline (PBS) were added to the cells for detecting the unfolded protein response (Samali et al. 2010a; Cawley et al. 2011). After 0 or 6 h, cells were washed once with 1 × PBS. Cells in plates were then harvested for RNA analyses according to the Promega RNA Cell extraction kit (Promega, Madison, WI) and cells in chamber slides were fixed with 4% paraformaldehyde (PFA; Histolab Products AB, Gothenburg, Sweden) for immunofluorescence staining.

### RNA extraction and qPCR analysis

RNA was also extracted from fibroblasts before and after addition of ER stress inducing chemicals (as described above). cDNA synthesis and quantitative real‐time PCR (qPCR) was performed as described previously (Tufvesson et al. 2011; Weidner et al. 2017). All *BiP, HERPUD1, ERP72, and WARS* mRNA expression was normalized against expression of the reference genes *β‐Actin* and *GAPDH* (for primer sequences see Table 2).

**Table 2:** Primer sequences

Primer name	Sequence
*β‐Actin*	5′ AGC ACA GAG CCT CGC CTT T
	3′ GGA ATC CTT CTG ACC CAT GC
*GAPDH*	5′ GAA GGT GAA GGT CGG AGT CA
	3′ TGG AGG ATG GTG ATG GGA TT
*GRP78/BiP*	5′ TAG CGT ATG GTG CTG CTG TC
	3′ TTT GTC AGG GGT CTT TCA CC
*HERPUD1*	5′ ACT TGC TTC CAA AGC AGG AA
	3′ CCC TTT GCC TTA AAC CAT CA
*ERP72*	5′ CAT CAA GGA CTT CGT GCT GA
	3′ TTC ACC TCC CCA GCA TAG TC
*WARS*	5′ CAC CCC TGA TTG GAC AGT CT
	3′ TGG AAG ACA CTG CAG AGG TG

### Immunofluorescence and microscopy

The staining procedure was performed as described previously (Tufvesson et al. 2017). Briefly, cells were rinsed with 1 × PBS and fixed with 4% PFA and rinsed before addition of permeabilization buffer (PBSTw; 1 × PBS; 1% Tween20) and blocked with blocking buffer (2% normal goat serum (NGS; Abcam, Cambridge, UK) in PBSTw). Cells were then incubated with various primary antibodies in blocking buffer. Cells were washed with PBSTw and incubated with fluorescently coupled secondary antibodies in blocking buffer. Cells were washed with 1× PBS and incubated with 30 nmol/L DAPI (Life Technologies, Carlsbad, CA) and rinsed in water before the addition of Dako mounting solution (DAKO, Glostup, Denmark) and a coverslip. Slides were stored in a dark, moist chamber at 4°C until observation. Microscopy was performed using a Nikon Eclipse 80i microscope (Nikon, Tokyo, Japan), equipped with an Olympus DP80 camera (Olympus, Tokyo, Japan), and a Nikon Plan Apo 40×/0.95 objective. Images were obtained with CellSens Dimension (Olympus) and processed in ImageJ software (U.S. National Institutes of Health, Bethesda, MD).

### Antibodies

Primary antibodies used included: protein disulfide isomerase (PDI, mouse anti‐human (Prod# MA3019); 1:250, Life Technologies), golgin‐97 (rabbit anti‐human (Prod #A21270); conc. 1:100, Life Technologies), and lysosomal‐associated membrane protein 1 (LAMP1, mouse anti‐human (ab25630); 1:100, Abcam Cambridge, MA). Secondary antibodies were: AlexaFluor488 (anti‐mouse (IgG1), conc. 1:750), AlexaFluor555 (anti‐mouse(IgG2a), conc. 1:750), and AlexaFluor647 (anti‐rabbit, conc. 1:750), all from Life Technologies.

### Active Caspase‐3 assay

Cells were grown to confluency in six well plates and processed and analyzed according to the protocol of the human Active Caspase‐3 Quantikine ELISA kit used (KM300, R&Dsystems, Minneapolis, MN).

### Statistics

Data were analyzed using Prism 5 software (Graphpad, La Jolla, CA) and used as described in the figure legends. *P* < 0.05 was considered significant. Kruskal–Wallis nonparametric test was used for analyses among several groups, and followed by Dunn's multiple comparison test for comparison between separate groups.

## Results

### Endoplasmic reticulum is disorganized in COPD patients

**Figure 1:**
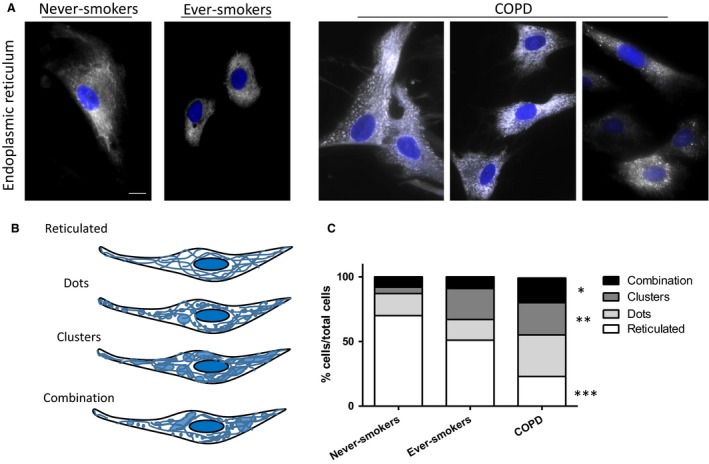
The endoplasmic reticulum is disorganized in Chronic Obstructive Pulmonary Disease (COPD) lung fibroblasts. Representative images of lung fibroblasts from never‐smokers and ever‐smokers are shown along with three different COPD patients. The endoplasmic reticulum (ER; white) appears more disorganized in COPD patients with a more dotty and patchy structure versus the more reticulated structure shown in never‐smokers and ever‐smokers (A). The nuclei are shown in blue. Phenotypes of the different ER structures (B) were counted and compared (C) for never‐smokers (*n* = 5), ever‐smokers (*n* = 5), and COPD patients (*n* = 12). Scale bar equals 20 *μ*m. *P < 0.05, **P < 0.01, and ***P < 0.001 showing significant differences among the subject groups in the proportion of the respective cell phenotypes.

In order to assess the effects caused by long‐term cigarette smoking on the internal membrane systems in the cell, we obtained lung fibroblasts from healthy never‐smokers, ever‐smokers, and COPD patients. We found the ER structure to be disorganized in COPD patients. Never‐smoking and ever‐smoking subjects had a reticulated network as was expected, but COPD patients showed a more clustered, punctate structure to varying degrees (Fig. 1A). To determine the level of disorganization seen in each patient, the number of cells showing reticulated, dotty, clustered, or a combination of the phenotypes were observed (Fig. 1B) and a minimum of 200 cells per subject group were counted. There was a significant difference between never‐smokers, ever‐smokers, and COPD patients (Fig. 1C), with a lower proportion of fibroblasts with reticulated ER in COPD patients (*P* = 0.0006) and instead a higher proportion of fibroblasts with clustered ER (*P* = 0.007) or the combination of dotty and clustered ER (*P* = 0.044), while the proportion of fibroblasts with a dotty phenotype was not significantly different (*P* = 0.19).

### Golgi and lysosomes are altered in COPD patients

**Figure 2:**
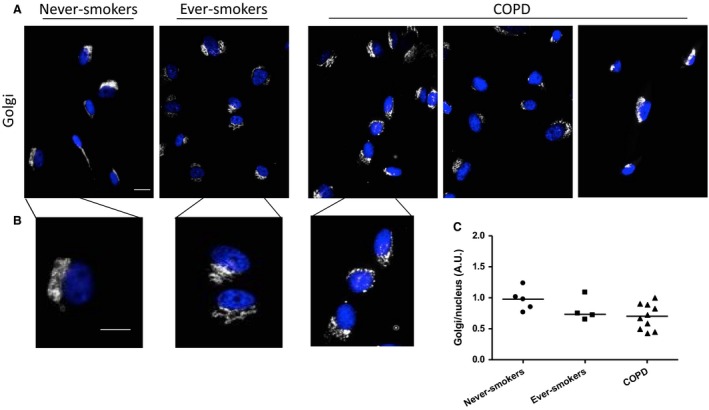
Golgi are altered in Chronic Obstructive Pulmonary Disease (COPD) lung fibroblasts. Representative images of Golgi in lung fibroblasts from never‐smokers, ever‐smokers, and three COPD patients are presented. The structure of the Golgi (white), overall, remained unchanged among the three groups (A), but measurements comparing Golgi size to nucleus size (C) showed a reduction in COPD patients (*n* = 10) compared to never‐smokers (*n* = 5) and ever‐smokers (*n* = 5). (C). Cells from (A) were enlarged to better show Golgi structure (B). Nuclei are shown in blue. Scale bar equals 20 *μ*m.

Due to the alterations seen in the ER of COPD patient lung fibroblasts, we examined other organelles in the endomembrane system. We found that in COPD patients, the size of the Golgi, as compared to the nucleus, was altered in lung fibroblasts as compared to never‐smokers or ever‐smokers, but not significant (*P* = 0.087, Fig. 2A–C).

**Figure 3:**
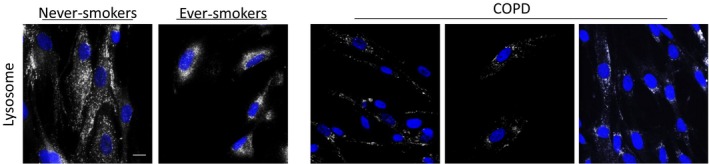
Lysosome distribution is changed in lung fibroblasts from smokers. Lysosomes (white) were examined in the three groups. In never‐smokers, lysosomes appeared distributed throughout the cell, more perinuclear in ever‐smokers, and dispersed, but less numerous in Chronic Obstructive Pulmonary Disease patients. Nuclei are shown in blue. Scale bar equals 20 *μ*m.

Furthermore, the lysosomes also had a different appearance. In never‐smokers, the lysosomes appeared numerous and found throughout the full‐length of the cell, whereas in ever‐smokers, the lysosomes were more clustered around the cell nucleus (Fig. 3). In COPD patients, lysosomes were spread throughout the cell, but did not seem as numerous as in never‐smokers.

### Internal membranes are altered in COPD patients under stress conditions

**Figure 4:**
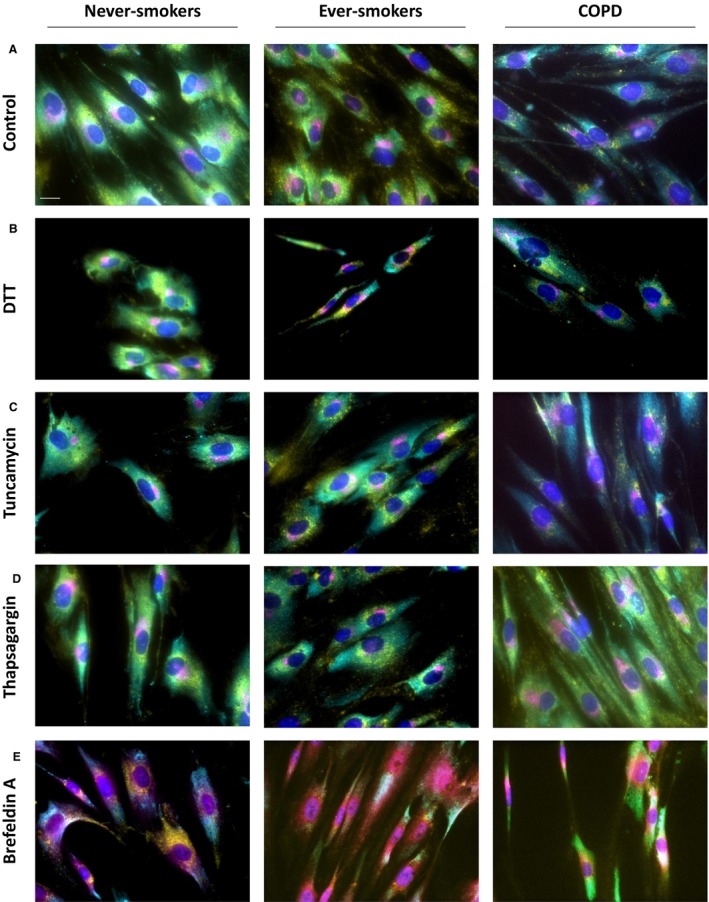
Endoplasmic reticulum (ER) stress induces changes in internal membranes. Time course experiments were performed in lung fibroblasts from never‐smokers, ever‐smokers, and Chronic Obstructive Pulmonary Disease patients with different ER stress‐inducing chemicals. Representative images of each subject and the control (A) or chemical treatments: DTT (B), tunicamycin (C), thapsigargin (D), and Brefeldin A (E) are shown. The ER (cyan), Golgi (magenta), lysosomes (yellow), and nuclei (blue) are depicted. Scale bar equals 20 *μ*m.

Due to the changes in the endomembrane system in COPD patients under normal culture conditions, we investigated if these cells would be sensitive to chemical stressors known to induce the ER stress response (Samali et al. 2010a; Fig. 4).

Under DTT stress, which results in unfolded proteins through the reduction of disulfide bonds (Jiang et al. 2015), we observed that fibroblasts from never‐smokers appeared to exhibit a change in the Golgi complex where it appeared to collapse into a ball‐like structure in the perinuclear region. This phenomenon was not readily observed in ever‐smokers or COPD patients (Fig. 4B).

Under stress from tunicamycin (Schonthal 2012), an inhibitor of N‐linked glycosylation (Huang et al. 2009), lysosomes appeared to change. In all cases, lysosomes appeared to cluster in a more perinuclear pattern. In ever‐smokers and COPD subjects, lysosomes also appear a bit more clustered, with larger patches occurring (Fig. 4C).

Thapsigargin (Schonthal 2012) is used to inhibit the sarco/endoplasmic reticulum Ca^2+^ ATPase. Treatment with thapsigargin resulted in COPD cells that were observed to have more spread out lysosomes, appearing throughout the cell, unlike their never‐smoker or ever‐smoker counterparts, which showed a tendency for perinuclear clustering (Fig. 4D).

Finally, Brefeldin A is known to disrupt the Golgi complex (Samali et al. 2010a) and this was observed in all cells. In both ever‐smokers and COPD patients, though, the appearance of lysosomes was less pronounced than in cells from never‐smokers (Fig. 4E).

### Expression of ER stress‐induced genes in COPD patients

**Figure 5:**
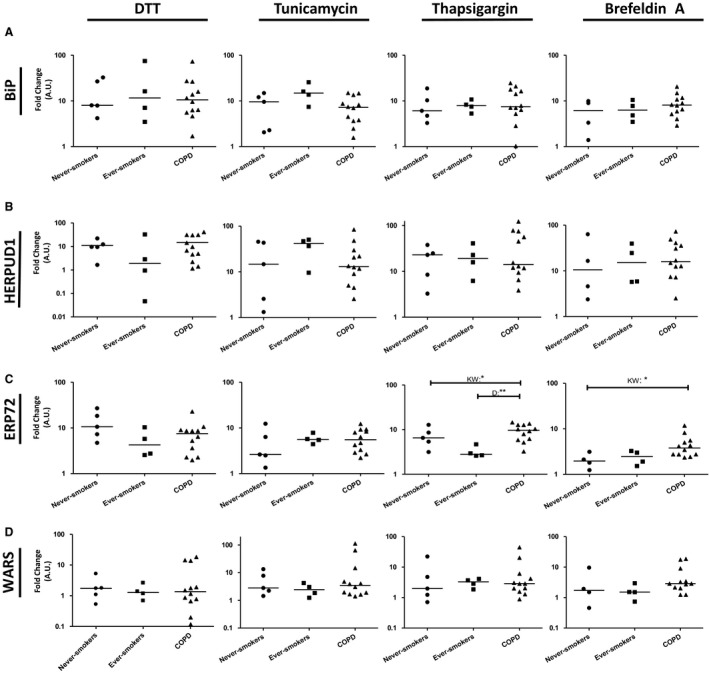
Changes of endoplasmic reticulum (ER) stress gene expression in Chronic Obstructive Pulmonary Disease (COPD) lung fibroblasts. The fold change of ER stress gene mRNA (the change between 0 and 6 h time point) in lung fibroblasts from never‐smokers (*n* = 5), ever‐smokers (*n* = 4), and COPD patients (*n* = 12) after ER stressors (DTT, tunicamycin, thapsagargin, and Brefeldin A) are presented. No major change in gene expression of *BiP* (A), *HERPUD1* (B), or *WARS* (D) among the different subject types or chemical stressors was observed. Nonsignificant changes to *ERP72* were observed with DTT and tunicamycin, but changes among the groups were observed in thapsigargin and Brefeldin A (C). The line in each case represents the median value. KW = Kruskal–Wallis, D = Dunn's multiple comparison test, *P < 0.05, **P < 0.01.

Since we observed phenotypic differences in never‐smoker, ever‐smoker, and COPD cells, we also investigated whether there were also changes in expression of genes induced under the ER stress, i.e., *BiP, HERPUD1, ERP72,* and *WARS* (Samali et al. 2010a), before and after addition of a stressor (fold change in expression compared to baseline is presented in Fig. 5). Overall, in most subjects there was an upregulation of ER stress response genes after addition of stressor compared to baseline, i.e., the fold change is >1 (Fig. 5). In addition, significant differences in stress response were seen between never‐smoker, ever‐smoker, and COPD cells in *ERP72* under stress by Brefeldin A and thapsigargin (Fig. 5C). No significant changes were observed in *BiP, HERPUD1*, or *WARS* expression after any stressor.

Additionally, we examined splicing of *XBP1* mRNA, which is induced upon ER stress (Cao and Kaufman 2014). mRNA from all subjects was analyzed at both 0 and 6 h during the time course. We observed *XBP1* splicing at 6 h in all cells in which chemical stressors were applied, but no splicing in any cells at 0 h. However, there was no difference between never‐smoker, ever‐smoker, and COPD cells (data not shown).

### Apoptosis in fibroblasts from smokers

**Figure 6:**
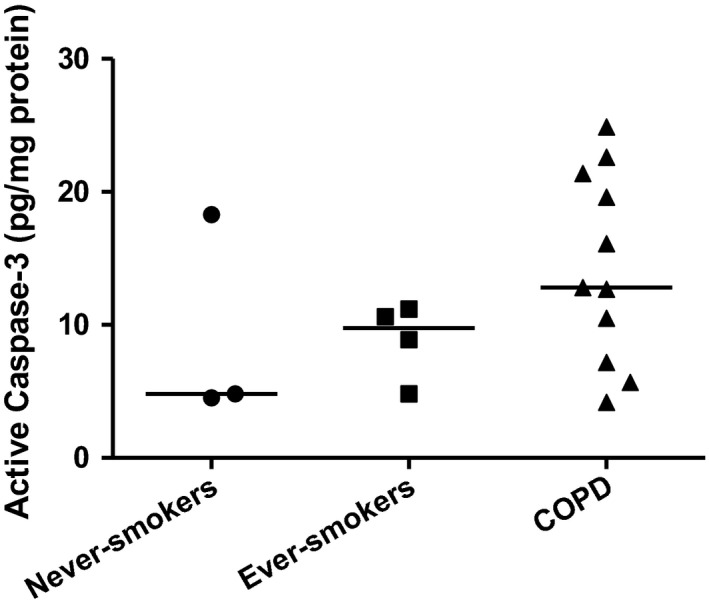
Active Caspase‐3 in lung fibroblasts. Levels of active Caspase‐3 were measured in lung fibroblasts from never‐smokers (*n* = 3), ever‐smokers (*n* = 4), and Chronic Obstructive Pulmonary Disease (COPD) patients (*n* = 11). A nonsignificant increase in active Caspase‐3 was observed in some COPD patients, but not in never‐smokers or smokers. The line in each case represents the median value.

To determine if apoptosis may be playing a role in the phenotypic changes seen in the lung fibroblasts, the concentration of active Caspase‐3 under unstressed conditions was determined (Sano and Reed 2013). When examining cells from never‐smokers, ever‐smokers, and COPD subjects, we found that there was an increase in active Caspase‐3 in some of the COPD patients, although this increase in the group as a whole was not significant (*P* = 0.28, Fig. 6).

## Discussion

We observed changes in the ER structure, Golgi size, and lysosome distribution in lung fibroblasts from COPD patients as opposed to the ever‐smokers and never‐smokers. In addition, we showed that fibroblasts from COPD patients were more sensitive to chemical stressors known to induce the ER stress response.

Stress is a physiological condition that all cells encounter at one point or another. We set out to examine if fibroblasts from COPD patients were more affected by the stress of continuous and prolonged smoking as opposed to their nonsmoking or non‐COPD counter parts. We found that even though fibroblasts show phenotypical differences in internal membranes among never‐smokers, ever‐smokers, and COPD patients, this phenotypic change did not inhibit their ability to respond to the ER stress response.

Not surprisingly, as the ER became less reticulated and more clusters and dotty structures were observed in COPD, the Golgi decreased in size as compared to the nuclear size. This change in Golgi size may point to a defect in protein trafficking from the ER to the Golgi complex. Likewise, a phenotypic change in the lysosomes of COPD patients was observed and the lysosomes present appeared smaller and/or less numerous than in never‐smokers or ever‐smokers, though this was difficult to quantify due to the difference in phenotypes among the three groups. For example, the clustered lysosome phenotype seen in ever‐smokers made distinguishing individual lysosomes extremely difficult for quantification purposes. These results would again suggest that there may be a defect in protein trafficking throughout the cell, resulting in less protein reaching their intended destination or less protein being transported overall. As fibroblasts are responsible for production and delivery of extracellular matrix components (Westergren‐Thorsson et al. 2010), the trafficking and/or composition of extracellular matrix proteins could be an interesting avenue for future research. Future studies to examine the trafficking dynamics of proteins traveling through the endocytic pathway would be crucial in better understanding of how the endomembrane system is affected in COPD patients.

The time course experiments were used to address the question: were fibroblasts from COPD patients able to be stressed and react in a manner similar to their non‐COPD counterparts? Some of the most obvious changes were observed in the Golgi and lysosomes of ever‐smokers and COPD patients. Drugs such as DTT and tunicamycin lead to unfolded and unmodified proteins that then need to be degraded or else they could be improperly trafficked in the cell (Schonthal 2012). In all subject groups, the lysosomes appear more perinuclear after addition of those drugs. This could perhaps be a cellular response to allow for accumulated proteins to be more quickly degraded in the lysosome. In both ever‐smokers and COPD patients, the lysosomes also appear to be morphologically larger than in controls, but this observation would have to be further quantified. An increase in lysosome size may also support increased degradation of proteins. Similarly, the Golgi collapsing into a ball‐like structure, could be the result of less proteins trafficking through the endocytic pathway. From our phenotypical study of stress to the endomembrane system, it appears that the Golgi is one of the most vulnerable organelles to stress. Further studies into specific trafficking of proteins could give more insight into how the Golgi responds to ER stress in the fibroblasts.

Previous studies on cellular stress in lung cells focused primarily on epithelial cells (Kuwano 2007; Geraghty et al. 2011; Ribeiro and O'Neal 2012; Somborac‐Bacura et al. 2013). Although epithelial cells are indeed the first line of defense against external stressors, the fibroblasts are one of the main extracellular matrix producers in the lungs and therefore of potential impact in the context of COPD development (Westergren‐Thorsson et al. 2010). To our knowledge, this is the first study of ER stress in human airway fibroblasts in response to stress.

Although we now know that fibroblasts from ever‐smokers and COPD patients are able to still respond to different forms of endoplasmic reticulum stress, it is still unclear exactly what the differences in phenotypes mean. Furthermore, we do not know how the cells would respond to an acute response such as addition of cigarette smoke extract. This would be an interesting area of interest for future studies.

Previous studies have focused on established cell lines (Somborac‐Bacura et al. 2013) or mouse experiments (Geraghty et al. 2011; Kenche et al. 2013; Lee et al. 2016), whereas we have used primary lung fibroblasts grown out from patient biopsies. These biopsies were taken and cells were cultured for several weeks before experiments were performed. Conditions such as this may affect the way in which the cells respond to stress as opposed to using fresh cells and an acute response. This may explain why we only see minor difference in ER stress response in our fibroblasts.

Regardless, we are able to see subtle, though nonsignificant differences among the different groups when it comes to the monitoring of ER stress response genes. In our study, we used four different genes known to be induced upon ER stress response although several other genes could have been used. *BiP/GRP78, HERPUD1, ERP72,* and *WARS* are common reporter genes for ER stress response (Samali et al. 2010a; Oslowski and Urano 2011; Schwarz and Blower 2016). These genes represent proteins needed to aid in the restoration of homeostasis to the cell and mark a functional representation of the IRE1 and ATF6 branches of the ER stress response pathways. In most subjects, that is never‐smokers, ever‐smokers and COPD patients, there was an upregulation of ER stress response genes after addition of the stressors compared to baseline. In addition, *ERP72* showed a different stress response between the different subject groups, suggesting that there is an altered response to stress in smoking COPD patients. Due to limited amount of material, we were unable to examine all the possible genes associated with ER stress and chose, instead, to focus on the genes often used to monitor stress response (Samali et al. 2010a,b; Oslowski and Urano 2011).

Additionally, the IRE1 arm of the ER stress response pathway was monitored by *XBP1* splicing (Cao and Kaufman 2014), which appeared normal among the three groups of subjects in this study and *XBP1* splicing was not observed under nonstress conditions (data not shown). The absence of an activated ER stress response under normal, nonstress conditions, indicates that despite the phenotypical changes to the endomembrane system, the signals for ER stress are not substantially activated. We did find, however, that when cells were dosed with stressors known to activate ER stress, all cells responded, regardless of subject group. Thus, despite seemingly chronic oxidative stress from cigarette smoking, COPD fibroblasts could activate the ER stress response at least as readily as their never‐smoking counterparts.

Even though there seemed to be internal membrane changes in COPD patient fibroblasts, we could not define an overall significant induction of apoptosis. If cellular stress cannot be alleviated, it is possible that this will trigger apoptosis in the cell (Sano and Reed 2013). We found that in some patients there was an increase in the amount of active Caspase‐3 under unstressed conditions, but this varied widely in COPD patients (Fig. 6).

The ever‐smokers and COPD patients were age‐matched, but the never‐smokers were slightly younger. This is a limitation of the study, but we believe that this is of only minor significance since most stress results depicted the ever‐smokers to be an intermediate between the never‐smokers and the age‐matched COPD patients. This is also a small observational study with only a small number of included subjects, nevertheless, these observations point to interesting new areas of COPD research on the molecular level.

We have begun to characterize an interesting cellular phenotype in central airway fibroblasts from never‐smokers, ever‐smokers, and COPD subjects; however, additional experimentation in regards to intracellular structure should be an avenue for future research. Previous studies using murine models have looked via electron microscopy into various cells from the lung (Mahavadi et al. 2014; Yu et al. 2015; Liu et al. 2017). Though, these studies did not include an in‐depth look into the different membrane structures. Mahavadi and colleagues used an antiarrhythmis drug reported to cause lung fibrosis in order to examine ER and lysosomal stress in the lung (Mahavadi et al. 2014). Using transmission electron microscopy to examine alveolar areas, they likened their images to those seen in Hermansky Pudluk syndrome (HPS), which is a disease caused by protein trafficking defects, something that might explain our disorganized endomembrane phenotypes. Different irritants have also been examined in the lung at the ultrastuctural level in terms of ER stress (Oh and Lim 2009; Yu et al. 2015). In both studies, swelling/ballooning of the ER in cultured normal human fibroblasts or lung tissue from mouse was observed in response to the stressor (Oh and Lim 2009; Yu et al. 2015). In addition, in a rat smoking model of COPD (Liu et al. 2017), it has been observed by scanning electron microscopy that the rats in the smoking COPD model, as compared to the control animals, had an expanded ER. This finding may show a similar phenotype to what we have seen in our COPD patients. Additionally, transmission electron microscopy has been used to explore the epithelial–mesenchymal transition in mild‐moderate COPD patients (Behzad et al. 2009). Although this study more focused on epithelial‐fibroblast contacts and the architecture in the lung, it would be interesting to look more in depth into fibroblasts in the context of the whole lung and not merely in vitro. Additionally, ultrastructural evaluation of the examined subjects would allow us to more accurately assess the level of disorganization in various endomembrane systems as we have observed alternations to all of these compartments in our study. It would be of great interest to determine how the internal structure is altered by electron microscopy and compare it to our findings by fluorescence microscopy.

In conclusion, we have found that ER, Golgi, and lysosomes in lung fibroblasts from COPD patients differ from those of never‐smokers and ever‐smokers. These changes suggest that protein trafficking through the COPD cells might be affected. In addition, difference in the phenotypical and genetic upregulation of stress response was most prominent in fibroblasts from most COPD patients. This demonstrates that the prolonged exposure to oxidative stress, such as that suggested to occur with cigarette smoke, may permanently alter the physical makeup of proteins within these organelles.

## Conflict of Interest

None of the authors have any conflict of interest.
